# Promoting Nrf2/Sirt3-Dependent Mitophagy Suppresses Apoptosis in Nucleus Pulposus Cells and Protects against Intervertebral Disc Degeneration

**DOI:** 10.1155/2021/6694964

**Published:** 2021-06-09

**Authors:** Sunli Hu, Chenxi Zhang, Tianchen Qian, Yue Bai, Liang Chen, Jiaoxiang Chen, Chongan Huang, Chenglong Xie, Xiangyang Wang, Haiming Jin

**Affiliations:** ^1^Department of Orthopaedics, The Second Affiliated Hospital and Yuying Children's Hospital of Wenzhou Medical University, Wenzhou, Zhejiang, China; ^2^The Second School of Medicine, Wenzhou Medical University, Wenzhou, Zhejiang, China; ^3^College of Basic Medical Sciences, Dalian Medical University, Dalian, China

## Abstract

One of the causes of intervertebral disc degeneration (IVDD) is nucleus pulposus cell (NPC) death, possibly apoptosis. In this study, we explored the role of the Nrf2/Sirt3 pathway and tert-butylhydroquinone (t-BHQ) in IVDD and elucidated the potential working mechanism. Reactive oxygen species (ROS) assay kits and malondialdehyde (MDA) assay kits were used to assess oxidative stress. Western blot and TUNEL staining were used to examine apoptosis. After siRNA against Nrf2 or lentivirus against Sirt3 was transfected into NPCs, the mechanism of the effect of the Nrf2/Sirt3 pathway on NPCs was assessed. The interaction between t-BHQ and its potential interacting protein NRF2 was further investigated through protein docking analysis. ChIP examined the binding affinity between Nrf2 and Sirt3 promoter. *In vivo* experiments, X-ray, hematoxylin-eosin (HE) staining, Safranin O staining, and immunohistochemistry were used to evaluate IVDD grades. The results demonstrated that activation of the Nrf2/Sirt3 pathway inhibited tert-butyl hydroperoxide- (TBHP-) induced apoptosis and mitochondrial dysfunction in vitro. In addition to apoptosis, upregulation of the Nrf2/Sirt3 pathway induced by t-BHQ restored TBHP-induced autophagic flux disturbances. However, its protective effect was reversed by chloroquine and Si-ATG5. Furthermore, t-BHQ ameliorated IVDD development in a rat model. In conclusion, our findings indicate that the Nrf2/Sirt3 pathway and its agonist represent a potential candidate for treating IVDD.

## 1. Introduction

Low back pain is one of the most common causes of disability in developed countries, and the number of affected patients is increasing worldwide [1]. This disease exerts an enormous socioeconomic burden on societies, families, and individuals. Effective interventions are still hampered because the many causes of low back pain are not well understood. A strong relationship between low back pain and intervertebral disc degeneration has been documented [2–4]. Unfortunately, the etiology of intervertebral disc degeneration is also unclear, and most investigators agree with the current idea that it is “multifactorial” in nature. There are three types of cells located in the intervertebral disc: (1) the gelatinous nucleus pulposus, (2) annulus fibrosus cells, and (3) endplate chondrocytes [5]. Among them, NPCs maintain biomechanical equilibrium and the rock-steady structure of the intervertebral disc. Oxidative stress, an important pathological factor, may produce excessive reactive oxygen species (ROS), potentially resulting in apoptosis [6]. Therefore, reducing apoptotic cells is an effective method to delay IVDD.

Sirtuin 3 (SIRT3), localized in the mitochondria, works as a deacetylase of mitochondrial protein [7–9]. SIRT3 maintains mitochondrial homeostasis by promoting mitochondrial dynamics, antioxidation, and mitophagy, as well as deacetylation of manganese superoxide dismutase (MnSOD), enhancing its biological activity in senescent NPCs and indicating that SIRT3 might play an important role in normal mitochondrial function in NPCs against oxidative stress [10].

tert-Butylhydroquinone (t-BHQ), an agonist of nuclear respiratory factor 2 (Nrf2), is an aromatic organic compound with highly effective antioxidant and anti-inflammatory functions, and it can restore dysfunctional mitochondria [11–13]. Recently, one study suggested that NRF2 is a regulator of SIRT3 expression and may shed light on how SIRT3 is upregulated during nutrient stress [14]. Since specific treatment of IVDD remains an unmet medical need, it is imperative to pursue effective strategies for the clinical management of IVDD. The present study is aimed at investigating the protective effects of t-BHQ in IVDD and at determining the potential therapeutic mechanisms.

## 2. Materials and Methods

### 2.1. Ethical Statement

All surgical interventions, treatments, and postoperative animal care procedures were performed in strict accordance with the Animal Care and Use Committee of Wenzhou Medical University. As for the human specimens, the study was authorized by the Ethical Committee of the Second Affiliated Hospital, Wenzhou Medical University, and followed the guidelines of the Declaration of Helsinki [15].

### 2.2. Reagents and Antibodies

tert-Butylhydroquinone (t-BHQ) and bafilomycin A1 were obtained from MCE (Monmouth Junction, NJ, USA). tert-Butyl hydroperoxide solution (TBHP), chloroquine (CQ), and type II collagenases were purchased from Sigma-Aldrich (St. Louis, MO, USA). Primary antibodies against BCL-2, Bax, SIRT3, NRF2, LC3, and collagen II were obtained from Abcam (Cambridge, MA, USA). Antibodies against GAPDH and p62 were acquired from Proteintech (Chicago, IL, USA). Antibodies against TOM20 and aggrecan were from Santa Cruz Biotechnology. The antibody against cleaved caspase-3 was from Affinity (Cincinnati, OH, USA). Alexa Fluor 488-labeled and Alexa Fluor 647-labeled goat anti-rabbit/rat secondary antibodies were purchased from Abcam (Cambridge, MA, USA). 4,6-Diamidino-2-phenylindole (DAPI) was obtained from Beyotime (Shanghai, China). Reagents for NPC culture were obtained from Gibco (Grand Island, NY, USA).

### 2.3. Clinical Study Population/Human Specimens

NP tissues with different degeneration grades (*n* = 9; age 22 to 68 years, mean age 35 years) were collected from patients who underwent disc resection or spinal fusion surgery.

### 2.4. Primary Rat Nucleus Pulposus Cell Culture

Nucleus pulposus tissues were isolated from twenty male Sprague-Dawley rats (4 weeks old) under sterile conditions. NP tissues were cut into pieces and incubated with 0.1% collagenase II in DMEM/F12 medium at 37°C for 4 h. After washing and suspension, NPCs were seeded into culture flasks in DMEM/F12 supplemented with 15% fetal bovine serum (FBS), 100 U/mL penicillin, and 100 *μ*g/mL streptomycin in 5% CO_2_ at 37°C. After 48 h of incubation, culture media were replaced with fresh media. When cell density reached 70% confluence, NPCs were harvested with 0.25% trypsin-EDTA and reseeded into six-well culture plates at the appropriate density. The next passage of cells was used for experiments and replenished with fresh media every 3 days.

### 2.5. siRNA and Lentivirus Transfection

When cultures reached 40–60% density, NPCs were transfected with lentivirus (GeneChem, China) for the SIRT3 gene and siRNA (Thermo Fisher Scientific, USA) for NRF2 and the ATG5 gene at a multiplicity of infection (MOI) of 100. After 8 hours of transfection, culture media were replaced with fresh media. When confluent, transfected NPCs were passaged for further experiments.

### 2.6. Cell Viability Assay

The cytotoxicity of t-BHQ on NPCs was evaluated with a Cell Counting Kit-8 (CCK-8) assay (Dojindo Co., Kumamoto, Japan). According to the manufacturer's protocol, NPCs were seeded into 96-well plates (5 × 10^5^ cells per well) and treated with different concentrations of t-BHQ according to the experimental design. Following treatment, cells were washed with PBS, and 10 *μ*L tetrazolium substrate was added into 90 *μ*L media of each well and incubated at 37°C for 1.5 hours. The absorbance of each well was measured at 450 nm using a microplate reader (Thermo Scientific, USA).

### 2.7. Western Blot Assay

Proteins were isolated from rat NPCs and lysed in ice-cold RIPA buffer with 1 mM PMSF (phenylmethanesulfonyl fluoride, Beyotime) followed by 10 min centrifugation at 12,000 rpm at 4°C. Then, protein concentrations of samples were measured by the BCA protein assay kit (Beyotime). Equivalent amounts of protein were separated on 8%-12% sodium dodecyl sulfate-polyacrylamide gel electrophoresis (SDS-PAGE) and transferred to polyvinylidene difluoride (PVDF) membranes (Millipore, USA) followed by blocking in 5% nonfat milk in TBST (Tris-buffered saline with 0.1% Tween-20) for 90 minutes at room temperature. Next, membranes were probed with primary antibodies overnight at 4°C. The next day, membranes were washed with TBST and incubated with respective secondary antibodies. Results were quantified using the Image Lab 3.0 software (Bio-Rad).

### 2.8. TUNEL Staining

Apoptosis was detected by the terminal deoxynucleotidyl transferase (TdT) dUTP nick end labeling (TUNEL) method (Roche, CA). After fixing in 4% paraformaldehyde for 0.5 h, NPCs were incubated with 3% H_2_O_2_ for 10 minutes and 0.1% Triton X-100 for 15 minutes. Cells were then washed with PBS 3 times and costained with TUNEL inspection fluid and 4′,6-diamidino-2-phenylindole (DAPI) according to the manufacturer's instructions. Finally, six random microscopic fields per slide were selected and captured under a Nikon ECLIPSE Ti microscope (Nikon, Tokyo, Japan).

### 2.9. Immunofluorescence

Following treatment, NPCs were washed in PBS, fixed in 4% paraformaldehyde for 15 minutes, and permeabilized in 0.1% Triton X-100 for 3 minutes. Next, cells were blocked in 5% bovine serum albumin for 1 h at 37°C and incubated with primary antibodies against Nrf2 (1 : 100), LC3 (1 : 100), and TOM20 (1 : 100) in a humid chamber overnight at 4°C. The next day, NPCs were washed and subsequently incubated with Alexa Fluor 488- or Alexa Fluor 594-conjugated secondary antibodies for 1 h at 37°C and labeled with DAPI for 5 min. Slides were observed with a Nikon ECLIPSE Ti microscope (Nikon, Tokyo, Japan).

### 2.10. Mitochondrial Membrane Potential Assay

Mitochondrial transmembrane potential (MMP) was assessed using a red fluorescent dye, MitoTracker Red CMXRos (Molecular Probes™, Thermo Fisher Scientific Inc.), which stains mitochondria in live cells at a concentration of 100 nM for 30 min at 37°C. Then, nuclei were stained with DAPI for 15 min at 37°C. Slides were observed in a Nikon ECLIPSE Ti microscope (Nikon, Tokyo, Japan), and fluorescence intensity was quantified using the ImageJ software 2.1 (Bethesda, MD, USA).

### 2.11. MitoSOX Assay

The level of intracellular superoxide anions was assessed by using a red fluorescent dye, MitoSOX (Life Technologies), which stains superoxide anions in cells and builds up in a superoxide anion-dependent manner at a concentration of 5 *μ*M for 45 min at 37°C according to the instructions. Next, nuclei were stained with Hoechst DAPI for 15 min at 37°C. Each sample was observed in a Nikon ECLIPSE Ti microscope (Nikon, Tokyo, Japan), and fluorescence intensity was quantified using the ImageJ software 2.1 (Bethesda, MD, USA).

### 2.12. Autophagy Flux Analysis

NPCs were grown on 6-well plates and reached 50–70% confluence at the time of infection. After three washes, cells were infected with Ad-mCherry-GFP-LC3B adenovirus (Beyotime Institute of Biotechnology, Shanghai, China) at a multiplicity of infection of 100 for 24 h. Following treatment, autophagy flux was assessed under a Nikon ECLIPSE Ti microscope (Nikon, Tokyo, Japan).

### 2.13. Flow Cytometry Analysis

After treatment, the cells were collected and washed with PBS. Following the standard steps of the instructions of the commercial kit, cells were resuspended with 500 *μ*L binding buffer and stained with 5 *μ*L of annexin V-FITC and PI at 37°C in the dark for 30 min. Finally, the cell apoptosis rate was detected by flow cytometry (BD Company, Franklin Lakes, NY, USA).

### 2.14. Assessment of Oxidative Stress

After treatment and washing with PBS 3 times, cells were incubated with reactive oxygen species (ROS) assay mixture according to the protocol of the reactive oxygen species (ROS) assay kit. Five random fields were observed and imaged per slide using a Nikon ECLIPSE Ti microscope (Nikon, Tokyo, Japan). The levels of malondialdehyde (MDA) were determined by using commercially available total superoxide dismutase and malondialdehyde assay kits (Nanjing Jiancheng Bioengineering Institute, China) according to the manufacturer's protocol. The MnSOD assay kit with WST-8 (Beyotime Institute of Biotechnology, Shanghai, China) was used to measure SOD2 enzymatic activity according to the manufacturer's instructions.

### 2.15. RT-qPCR

The PCR experiment was conducted according to the previous established experimental method. The total RNA of cells was extracted using TRIzol. Complementary DNA was synthetized and then amplified using the PrimeScript-RT reagent kit and SYBR Premix Ex Taq (Sangon). Levels of target genes were analyzed using the 2^–ΔΔct^ method.

### 2.16. Puncture-Induced Rat Intervertebral Disc Degeneration Model

Thirty adult male SD rats (200–250 g) used for our study were randomly divided into three groups (sham, IVDD, and t-BHQ+IVDD). Rats were maintained in a controlled environment under standard conditions of temperature and humidity and an alternating 12 h light and dark cycle. The experimental region of the rat tail disc (Co5-6, Co8-9) was located by using an X-ray radiograph. The most widely accepted IVDD rat model was utilized in this study [16]. Rats were anaesthetized by intraperitoneal injection of 2% pentobarbital (40 mg/kg). Using an aseptic technique, a sagittal skin incision was created to expose the Co5-6 and Co8-9 disc, which was then punctured using a 30-gauge syringe as previously described [17]. The needle was inserted straight into the nucleus pulposus of the tail without damaging the endplate as observed under X-ray. The needle was then rotated through 360° and held in position for 1 minute. After surgery, rats in the t-BHQ group were immediately intraperitoneally injected with t-BHQ (50 mg/kg) and every 3 days thereafter until they were sacrificed. The other rats were injected with the same amount of saline every 3 days. Two months after surgery, rats were sacrificed with an overdose of phenobarbital according to the AVMA Guidelines for the Euthanasia of Animals.

### 2.17. X-Ray Image Acquisition

Eight weeks after puncture injury, X-ray images were taken on all animals. All rats were anaesthetized by intraperitoneal injection of 10% pentobarbital (40 mg/kg). Rats were fixed in a prone position for X-ray imaging by using an X-ray irradiation system (Kubtec). The disc height index (DHI) was assessed using a previously published method [18]. Alterations in the DHI of the punctured IVDs were expressed as %DHI (%DHI = postpunctured DHI/prepunctured DHI × 100%).

### 2.18. Histopathological Analysis

Rats were euthanized 8 weeks after puncture injury by administration of intraperitoneal overdose of 4% pentobarbital. The experimental regions of the disc, including the injured segment and nonpunctured tail, were collected for further analysis. Tissues were fixed in 4% paraformaldehyde, decalcified with 10% ethylenediaminetetraacetic acid solution, and paraffin-embedded for the serial section. Slides of the disc were stained with hematoxylin and eosin (H&E) and Safranin O-fast green (S-O) staining to determine changes in cellularity and morphology of NP and AF microscopically. Results were examined by three experienced histology researchers in a blinded manner. The histological score was assessed based on changes of histological appearance of the NP and AF [19, 20]. All discs were classified as normal, moderately degenerated, or severely degenerated according to the histological grading scale (the histological score of normal was 5; moderate degeneration, 6 to 11; and severe degeneration, 12 to 14).

### 2.19. Immunohistochemistry

After deparaffinization, each section was incubated in 3% H_2_O_2_ for 10 min and washed in PBS. Then, sections were incubated with 0.1% trypsin for 20 min at 37°C and washed with PBS. Sections were blocked in 1% goat serum albumin for 1 h at 37°C without PBS washing. Sections were then incubated with a primary antibody against cleaved caspase-3 (1 : 200) and LC3-II (1 : 200) at 4°C overnight. The next day, the sections were washed in PBS twice and incubated with HRP-conjugated secondary antibodies for 1 h at room temperature. At least three sections from each specimen were observed under a microscope (Olympus Inc., Japan). The rate of positive cells in each section was quantitated by the ImageJ software 2.1 (Bethesda, MD, USA).

### 2.20. Chromatin Immunoprecipitation Analysis and qPCR Detection

ChIP assays were performed using a ChIP kit (Abcam 500) according to the manufacturer's protocol. ChIP-qPCR was performed using the Green Master Mix (BIORAD 1725270). 2-4 *μ*g of antibody (anti-Nrf2 or normal IgG) was added. Primers that targeted the promoter of Sirt3 were also used. Finally, digested chromatin was analyzed by quantitative real-time PCR.

### 2.21. Molecular Modeling

The protein-protein complex containing Nrf2 (PDB ID: 3WN7) was utilized to perform docking studies, which were obtained from PDB (https://www.rcsb.org/). After being minimized with PyMoL (version 1.7.6), the lowest energy conformations for docking were determined through default parameters. Protein-ligand docking analysis was conducted using the AutoDockTools (version 1.5.6). Images were generated with the UCSF PyMoL, and 2D interactions were plotted using Ligplus v.1.4.

### 2.22. Statistical Analysis

Results are expressed as the mean ± SD. Raw statistical analyses were processed using GraphPad Software, version 8.0.0 for Windows (San Diego, California, USA, http://www.graphpad.com/. Data were analyzed by one-way analysis of variance (ANOVA) followed by Tukey's test for comparison between different groups.

## 3. Results

### 3.1. Decreased Expression of Nrf2 in Human and Rat NPCs in IVDD and the Effect of t-BHQ on NPC Viability

**Figure 1:**
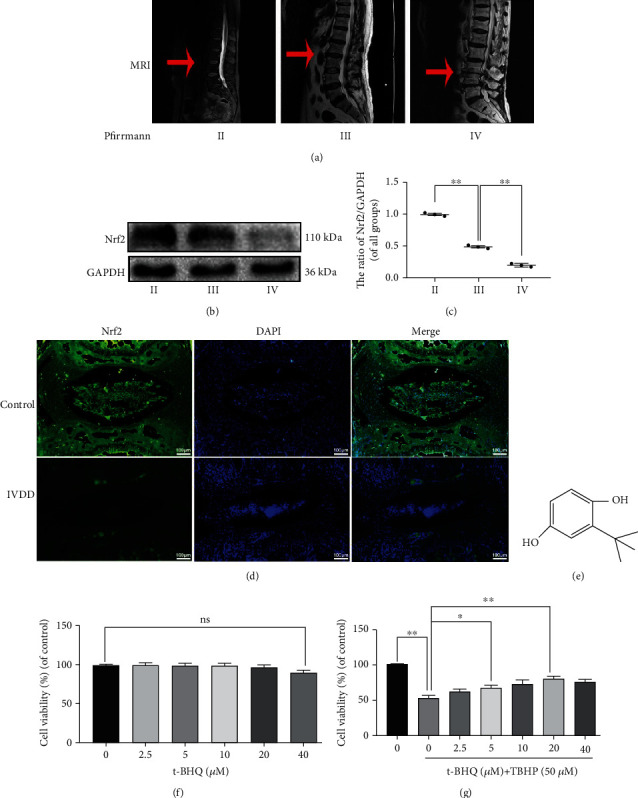
Decreased expression of NRF2 in human and rat NPCs in IVDD as well as effect of t-BHQ on NPC viability. (a) Representative MRI images of three different degrees of IVDD patients. (b, c) The expression of Nrf2 from NP cells of different degrees of IVDD patients was analyzed by western blot. (d) Immunofluorescence staining of Nrf2 in the disc of control and IVDD rat tail. (e) Chemical structure of t-BHQ. (f) The cytotoxic effect of t-BHQ on NPCs was measured at various concentrations for 24 h through using the CCK-8 assay. (g) The effect of t-BHQ against cell damage induced by TBHP in NPCs. All experiments were performed three times in duplicate, and data are reported as the mean ± standard deviation. ^ns^*p* > 0.05, ^∗^*p* < 0.05, and ^∗∗^*p* < 0.01.

NP tissue samples from IVDD patients were divided into three categories based on grade assigned according to a well-recognized classification system [21] (Figure 1(a)). Expression of Nrf2 in human NP tissue decreased with the increasing degeneration level (Figures 1(b) and 1(c)). The same result was observed in rat NP tissue. Immunofluorescence intensity of rat NP tissue for Nrf2 was decreased in NP tissue that had received puncture injury (Figure 1(d)). These results show that levels of Nrf2 are correlated with the severity of IVDD. To evaluate the toxicity of t-BHQ on NPCs, we performed a CCK-8 assay to assess the viability of NPCs treated with different concentrations of t-BHQ. The chemical structure of t-BHQ is shown in Figure 1(e). Results revealed that the appropriate concentrations of t-BHQ were 20 and 40 *μ*M, which did not impact cell viability (Figure 1(f)). We also found that t-BHQ reversed the loss in cell viability observed in response to TBHP stimulation in a dose-dependent manner. However, the higher concentration of t-BHQ did not show a better ability to improve cell viability (Figure 1(g)). Thus, we used 20 *μ*M as the experimental concentration in subsequent experiments.

### 3.2. t-BHQ Suppresses Apoptosis and Oxidative Injury Induced by TBHP in NPCs

**Figure 2:**
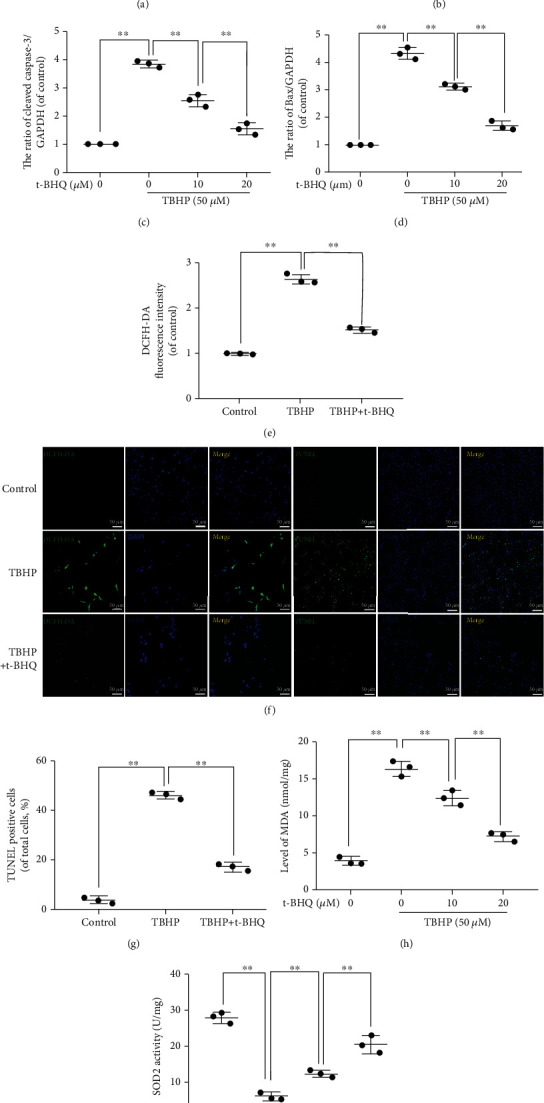
t-BHQ suppresses apoptosis and oxidative injury induced by TBHP in NPCs. (a) The expression of Bax, Bcl-2, and cleaved caspase-3 from different concentrations of t-BHQ-treated NPCs under TBHP stimulation for 24 h was analyzed by western blot. (b–d) Quantification of Bax, Bcl-2, and cleaved caspase-3 immunoblots. (e–g) TUNEL and ROS assays were performed in NPCs treated with t-BHQ and TBHP treatment for 24 h. (h) Intracellular MDA level was assessed by using the MDA assay kit. (i) Intracellular SOD2 level was assessed by using the SOD2 assay kit. All experiments were performed three times in duplicate, and data are reported as the mean ± standard deviation. ^∗^*p* < 0.05, ^∗∗^*p* < 0.01.

Given the relationship between Nrf2 and IVDD, as a Nrf2 agonist [22, 23], we wanted to determine whether t-BHQ alleviates cell damage in NPCs. To investigate the role of t-BHQ in apoptosis and oxidative stress in response to TBHP treatment, we performed several experiments. The TUNEL assay was performed, and protein expression of Bax, Bcl-2, and cleaved caspase-3 was assessed to assess apoptosis. To explore the effect of t-BHQ on TBHP-induced oxidative injury, we measured levels of ROS, MDA, and SOD2 activity in treated NPCs. For western blot results, upregulated cleaved caspase-3 and Bax levels were reversed after treatment with t-BHQ, while Bcl-2 changes were the opposite (Figures 2(a)-2(d)). For oxidative stress, DCFH-DA, accumulating in a ROS-dependent manner, is a special measurement to assess ROS. The higher fluorescence intensity representing DCFH was inhibited by t-BHQ (Figures 2(e) and 2(f)). In addition, increased TUNEL positivity induced by TBHP was decreased after administration of t-BHQ (Figures 2(f) and 2(g)). We also performed flow cytometry experiment, and the result was in accord with TUNEL (Figure S1). overexpression activates autophagy in severalInterestingly, pretreatment with t-BHQ reversed the trends observed in TBHP-induced oxidative injury.

### 3.3. t-BHQ Improves Nrf2's Activation and Antiapoptosis Effects

**Figure 3:**
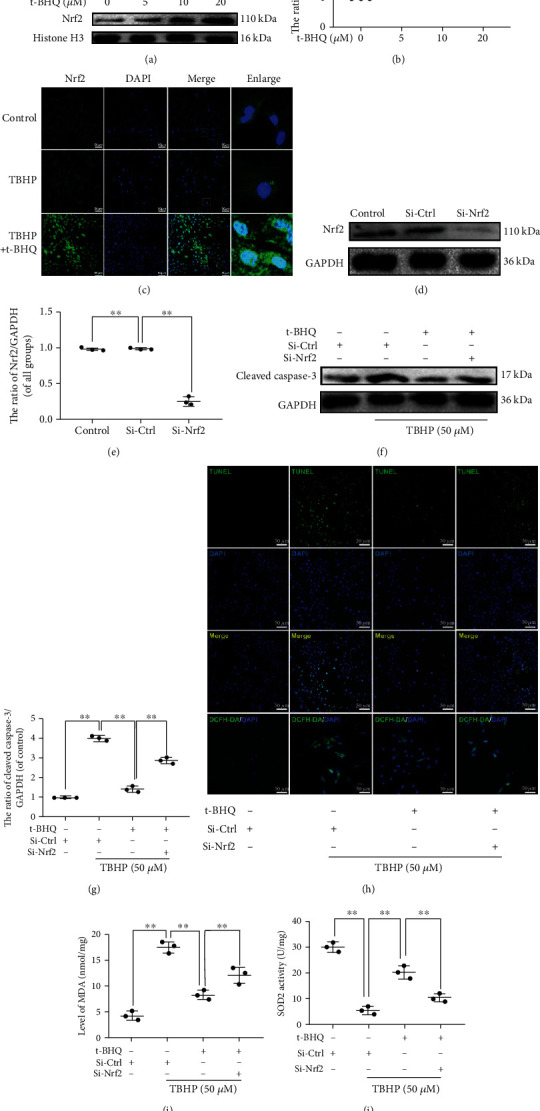
t-BHQ improves the capacity of antiapoptosis via NRF2 activation. (a, b) The expression of Nrf2 from different concentrations of t-BHQ-treated NPCs. (c) Representative images of Nrf2 in NPCs with t-BHQ and TBHP treatment for 24 h. (d, e) Successful knockdown of Nrf2. (f, g) The expression of cleaved caspase-3 in NPCs. (h) TUNEL and ROS assays were performed in NPCs. (i) Intracellular MDA level was assessed by using the MDA assay kit. (j) Intracellular SOD2 level was assessed by using the SOD2 assay kit. All experiments were performed three times in duplicate, and data are reported as the mean ± standard deviation. ^∗^*p* < 0.05, ^∗∗^*p* < 0.01.

To assess the capacity of t-BHQ to activate Nrf2 in NPCs, we treated NPCs with different concentrations of t-BHQ and extracted nuclear protein to perform western blot. Western blotting results demonstrated that t-BHQ increases Nrf2 nuclear expression in a dose-dependent manner (Figures 3(a) and 3(b)). NPCs treated with t-BHQ (20 *μ*M) under TBHP stimulation were subjected to immunofluorescence, and we found that t-BHQ facilitates Nrf2 puncta translocation into the nucleus compared to other groups (Figure 3(c)). As shown in Figures 3(d) and 3(e), primary rat NPCs transfected with Nrf2-knockdown siRNA (Si-Nrf2) displayed lower levels of Nrf2 compared to the scrambled siRNA group (Si-Ctrl). t-BHQ downregulated cleaved caspase-3 levels and TUNEL puncta in response to TBHP-induced oxidative stress, and these effects were partly reversed by Nrf2 knockdown (Figures 3(f)–3(h)). Moreover, for intracellular oxidative stress, our results suggested that Nrf2 knockdown suppressed t-BHQ-induced antioxidative effects through promoting higher levels of ROS and MDA and reduced SOD2 (Figures 3(h)–3(j)). Together, these results demonstrate that t-BHQ rescued TBHP-induced apoptosis and oxidative stress by promoting Nrf2 translocation into the nucleus.

### 3.4. Molecular Docking

**Figure 4:**
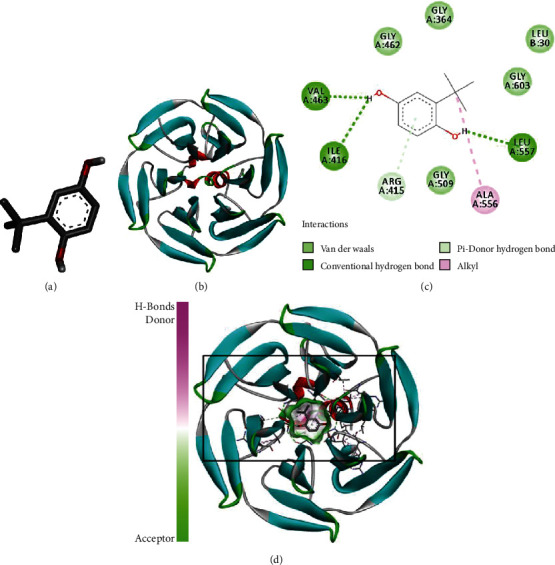
t-BHQ was docked with the Nrf2 structure. Docking studies were performed as described in Materials and Methods: (a) the model of STA; (b) the ribbon model of the Nrf2 protein-protein complex; (c) 2D binding model between the t-BHQ and Nrf2 complex; (d) 3D docking analysis between the t-BHQ and Nrf2 complex.

To examine whether t-BHQ has direct affinity for Nrf2 in NPCs, we constructed a molecular docking analysis. The chemical structure of t-BHQ and Nrf2 is shown in Figures 4(a) and 4(b). Results of the 2D binding model suggest that there are many interactions, including van der Waals, hydrogen bonds, and alkyl between t-BHQ and Nrf2 (Figure 4(c)). Furthermore, the 3D docking model revealed that t-BHQ can dock with Nrf2, showing a strong affinity at -12.5 kcal/mol (Figure 4(d)).

### 3.5. t-BHQ Protects NPCs from Cell Damage Induced by TBHP via Activation of Autophagy

**Figure 5:**
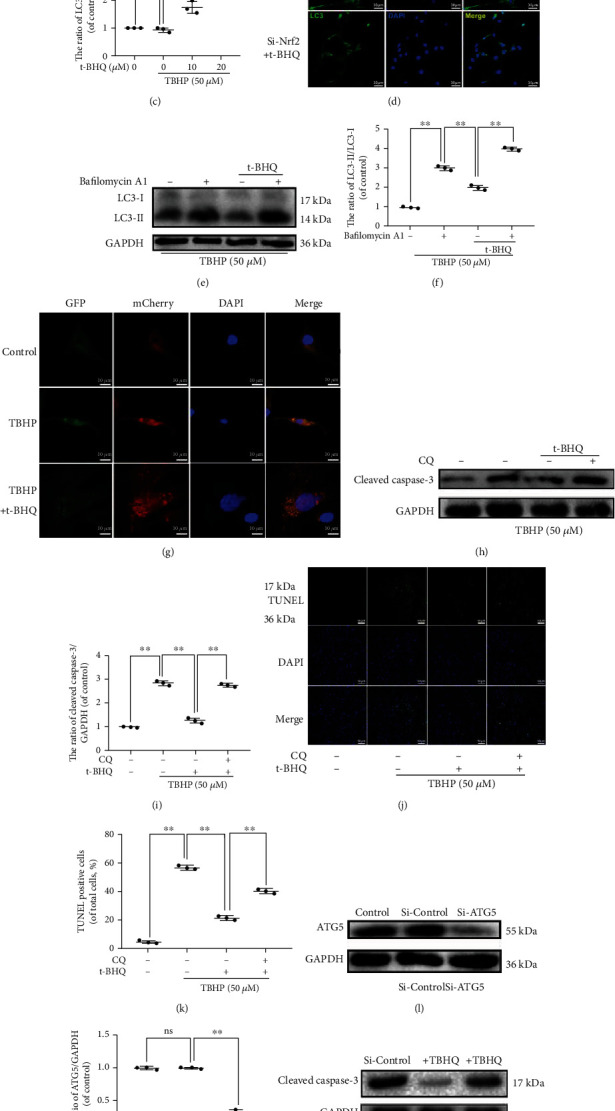
t-BHQ protects NPCs from cell damage induced by TBHP via activation of autophagy. (a–c) The expressions of LC3-I, LC3-II, and P62 in NPCs treated with different concentrations of t-BHQ and TBHP for 24 h. (d) Representative images of LC3 in NPCs. (e, f) The expressions of LC3-I and LC3-II in NPCs. (g) Representative images of immunofluorescent NPCs expressing mRFP-GFP-LC3. Autophagosomes (yellow dots in merged images) and autolysosomes (red only dots in merged images). Green fluorescence represents GFP, and red fluorescence represents mCherry. (h, i) The expressions of cleaved caspase-3 in NPCs. (j, k) TUNEL was performed in NPCs. (l, m) The expressions of ATG5 in NPCs treated with Si-control and Si-ATG5. (n, o) The expressions of cleaved caspase-3 in NPCs. All experiments were performed three times in duplicate, and data are reported as the mean ± standard deviation (*n* = 3). ^∗^*p* < 0.05, ^∗∗^*p* < 0.01.

Previously, we found that Nrf2 overexpression activates autophagy in several diseases [24, 25]. Therefore, we assessed whether t-BHQ promotes autophagy in NPCs in response to TBHP stimulation. We treated NPCs with different concentrations of t-BHQ in the presence of TBHP and found that LC3-II expression was increased in a dose-dependent manner, while P62 expression was decreased in t-BHQ groups (Figures 5(a)–5(c)). Furthermore, fluorescence staining results also showed that autophagy was activated (Figure 5(d)). Autophagic flux has been widely postulated as a potential therapeutic direction for IVDD. Our study also found that t-BHQ restores autophagic flux disturbed by TBHP pretreated with bafilomycin A1 (a typical lysosomal inhibitor). Expression levels of LC3-II among different NPC groups treated and untreated with Baf A1 were used to quantify autophagic flux (Figures 5(e) and 5(f)). In our previous work, we also determined that TBHP treatment blocks autophagic flux [26]. To monitor autophagic flux, tandem fluorescent mCherry-GFP-LC3B was performed on NPCs. Under normal situation, NPCs exhibited basal autophagy with few autophagosomes and autolysosomes. In the t-BHQ-treated group, NPCs possessed more autophagosomes and autolysosomes compared with those in other two groups, indicating that t-BHQ treatment additionally enhanced autophagic flux (Figure 5(g)). To determine the effect of healthy autophagic flux in NPCs treated with t-BHQ under TBHP, we chose CQ, a classical lysosomal activity alkalizer, to inhibit autophagic flux. As shown in Figures 5(h)-5(k), protection of t-BHQ against apoptosis was markedly attenuated, as exhibited by western blot results of cleaved caspase-3 and the percentage of TUNEL-positive points. In addition to chloroquine, we used Si-RNA against AGT5, a nonpharmacological approach, to interfere with the autophagy process. Results indicated that there was no off-target effect in our results (Figures 5(l) and 5(m)). Furthermore, administration of Si-ATG5 abolished the antiapoptosis effect induced by t-BHQ (Figures 5(n) and 5(o)). These results suggest that t-BHQ induces antiapoptosis effects via regulation of autophagy.

### 3.6. t-BHQ Alleviates the Imbalance in Mitochondrial Dynamics Induced by TBHP and Promotes Mitophagy in a Nrf2/SIRT3 Pathway-Dependent Manner

**Figure 6:**
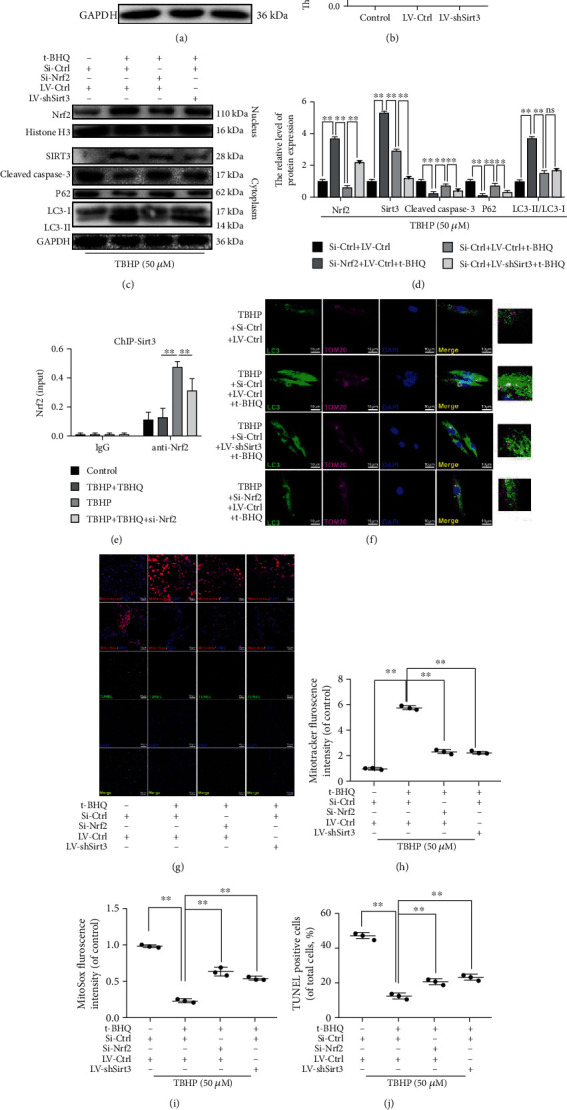
t-BHQ alleviates the imbalance of mitochondrial dynamics induced by TBHP and promotes mitophagy in a NRF2/SIRT3 pathway-dependent manner. (a, b) Successful knockdown of Sirt3. (c, d) The protein expression of LC3, P62, NRF2, histone H3, SIRT3, and cleaved caspase-3 in NPCs. (e) The promoter of Sirt3 was ChIP-ed with anti-Nrf2 antibody or IgG control. (f) Immunofluorescence double-labeled staining for colocalization of LC3 with TOM20 in NPCs. (g–j) TUNEL, MitoTracker, and MitoSOX staining assays were performed in NPCs. All experiments were performed three times in duplicate, and data are reported as the mean ± standard deviation. ^ns^*p* > 0.05, ^∗^*p* < 0.05, and ^∗∗^*p* < 0.01.

To explore the potential mechanism of the signaling pathway, we constructed a lentivirus to knockdown Sirt3 (Figures 6(a) and 6(b)). As shown in Figures 6(c) and 6(d), t-BHQ increased SIRT3 expression, which was decreased after administration of Si-Nrf2 as demonstrated by western blot. Interestingly, expression of Nrf2 was not altered in response to administration of LV-shSirt3, indicating that Sirt3 is downstream of NRF2. To measure the binding of NRF2 to Sirt3 promoters, ChIP was taken to examine the binding affinity. We found that there was higher affinity of Nrf2 with the Sirt3 promoter in the TBHP-treated group. Interestingly, administration of TBHQ further enhanced the expression of Nrf2 in Sirt3 promoter areas, whereas siRNA against Nrf2 reversed its upregulation (Figure 6(e)). For mitophagy status, autophagosomes were stained with LC3, and mitochondria were stained with the mitochondrial outer membrane marker Tom20. Immunofluorescence double staining results showed that increased formation of LC3-positive autophagosomes (green) was colocalized with the mitochondria (red) in t-BHQ-treated groups. However, the intensity of LC3 and colocalization of LC3 and TOM20 were markedly decreased after Sirt3 knockdown by lenti-shSirt3 (Figure 6(f)). From the western blot data, we observed that TBHP stimulation led to increased P62 and LC3-II levels, whereas t-BHQ further increased LC3-II and decreased P62, which was inhibited by Nrf2 and Sirt3 knockdown. Mitochondrial membrane potential is a marker for mitochondrial homeostasis. t-BHQ restored the imbalance of mitochondrial dynamics induced by TBHP, and this effect was reversed by Sirt3 and Nrf2 knockdown, as shown in the results of MitoTracker Red and MitoSOX assays (Figure 6(g)–6(i)). Furthermore, t-BHQ-induced antiapoptosis effects were inhibited when Sirt3 and Nrf2 were knocked down, as shown by western blot and TUNEL staining (Figures 6(g) and 6(j)).

Taken together, these results indicate that Sirt3 is a crucial component in t-BHQ-activated mitophagy, which mediates the clearance of damaged mitochondria and antiapoptosis effects in NPCs.

### 3.7. t-BHQ Ameliorates Intervertebral Disc Degeneration In Vivo

**Figure 7:**
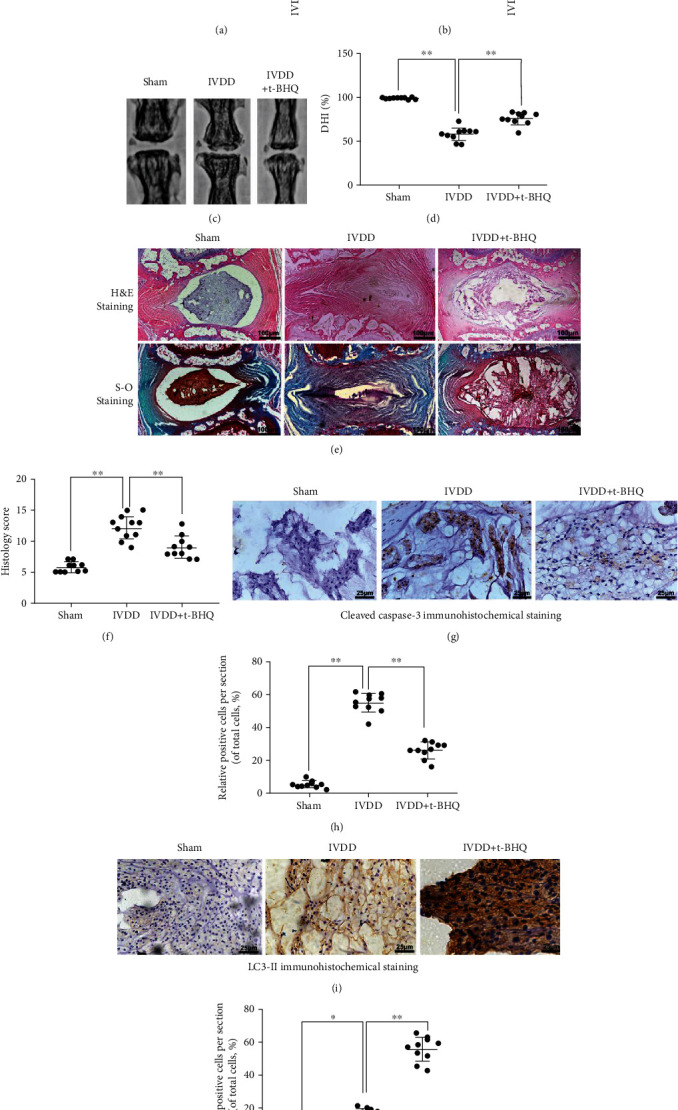
t-BHQ ameliorates intervertebral disc degeneration in vivo. After 8 weeks, degenerated discs were taken for X-ray and stained with H&E, Safranin O, and immunohistochemistry staining. (a) The mRNA expression of Nrf2 in nucleus pulposus tissue of punctured discs in different groups. (b) The mRNA expression of Sirt3 in nucleus pulposus tissue of punctured discs in different groups. (c, d) The disc height index (DHI) was determined in three groups at week 8. (e) Representative H&E and S-O staining of the punctured disc in different groups. (f) The histological grades in three groups. (g, h) The expression of cleaved caspase-3 was evaluated by immunohistochemistry staining in punctured discs in different groups. (i, j) The expression of LC3-II was evaluated by immunohistochemistry staining in punctured discs in different groups. All experiments were performed three times in duplicate, and data are reported as the mean ± standard deviation. ^∗^*p* < 0.05, ^∗∗^*p* < 0.01.

We constructed a rat IVDD model by puncture induction to evaluate the effects of t-BHQ on IVDD in vivo. To determine the role of T-BHQ in activating NRF2 and SIRT3, we measured Nrf2 and Sirt3 mRNA levels in NP tissue 8 weeks after the first intraperitoneal injection of t-BHQ and puncture surgery. Nrf2 and Sirt3 mRNA in the IVDD+t-BHQ group was significantly higher than that in the other two groups (Figures 7(a) and 7(b)). Radiographic images were obtained at 8 weeks, and we found that t-BHQ administration delayed the marked loss in disc height induced by puncture injury according to the X-ray results (Figures 7(c) and 7(d)). Hematoxylin and eosin staining was used to evaluate morphological changes. Safranin O staining was applied to assess proteoglycans and glycosaminoglycans of the intervertebral disc after t-BHQ treatment. Loss of NPCs, proteoglycans, and glycosaminoglycans was observed in the IVDD group, as well as lamellar disorganization or fragmentation. However, these changes were alleviated by t-BHQ treatment (Figure 7(e)). In addition, histological scores of the IVDD+t-BHQ group were significantly lower than those of the IVDD group at 8 weeks (Figure 7(f)). Apoptosis in NPCs was determined by cleaved caspase-3 immunohistochemical staining, and we found that the increased positive points of cleaved caspase-3 induced by puncture injury were reversed by t-BHQ treatment (Figures 7(g) and 7(h)). For assessment of autophagy in vivo, the result showed that t-BHQ significantly improved LC3-II level (Figures 7(i) and 7(j)). In conclusion, t-BHQ has therapeutic potential for alleviating the progression of IVDD.

## 4. Discussion

Various risk factors have been suggested to be associated with apoptosis and the imbalance of anabolic and catabolic activities, which accelerate development of IVDD [27]. Although there are ways to achieve early diagnosis, current treatments are used to relieve symptoms, such as low back pain and limb paralysis, and there is no effective treatment to slow the progression of the IVDD or to treat spinal disability. Although the pathophysiological mechanisms of IVDD are not entirely understood, inflammation and production of ROS impact NPC survival and have been implicated in IVDD's pathogenesis [28–30]. In this study, we used tert-butyl hydroperoxide (TBHP) as an ROS donor to induce oxidative stress, which led to apoptosis and mitochondrial and autophagy dysfunction characterized by blocked autophagic flux. We found that pretreatment with t-BHQ, an agonist of Nrf2, alleviates TBHP-induced oxidative stress in vitro and mitigates progression of IVDD. Moreover, t-BHQ also restored blocked autophagic flux and mitochondrial function. Furthermore, TBHP-induced apoptosis and matrix degradation were inhibited.

Nrf2, an important transcription factor belonging to the E26 transformation-specific (ETS) factor family, regulates cellular resistance to oxidative stress in several disease models, including osteoarthritis and intervertebral disc degeneration [31] [32, 33]. t-BHQ has been shown in many studies to protect cells from damage by promoting Nrf2's translocation to the nucleus to upregulate transcription of a range of protective genes [34, 35]. High ROS levels activate tyrosine kinases to block the Nrf2: Keap1 complex, nuclear import of Nrf2, and coordinated activation of cytoprotective gene expression. Additionally, Nrf2 activates antioxidant response element- (ARE-) mediated gene expression involved in cellular protection against oxidative stress. Therefore, we hypothesized that t-BHQ also has a protective effect in nucleus pulposus cells. As expected, administration of t-BHQ effectively alleviated apoptosis of nucleus pulposus cells induced by TBHP, and Si-Nrf2 weakens the therapeutic effect of t-BHQ.

Autophagy, a complex protein degradation system, is another strictly regulated and complex cellular process that works as a cellular quality control system to degrade damaged proteins or organelles [36–38]. Low nutrient levels and pathological factors, such as oxidative stress, can also activate autophagy, leading to restoration of normal metabolism and recovering cellular homeostasis through degradation of macromolecules [39]. One study recently demonstrated a direct interaction between p62 (an autophagy adaptor protein) and Keap1 (the Nrf2 substrate adaptor for the Cul3 E3 ubiquitin ligase) [40]. Trehalose promotes nuclear translocation of Nrf2 in a p62-dependent manner and increases expression of its downstream antioxidant factors, heme oxygenase-1 (Ho-1) and nicotinamide adenine dinucleotide phosphate quinone dehydrogenase 1 (Nqo1), which induce autophagy [41]. These studies reveal that Nrf2 suppressed cellular damage by activating autophagy. Thus, to investigate whether t-BHQ induces autophagy in NPCs to remit TBHQ-induced apoptosis, we pretreated NPCs with CQ, an autophagy inhibitor that inhibits the fusion of autophagosomes and lysosomes. Results showed that CQ reversed the antioxidative stress and antiapoptosis effects of t-BHQ. Levels of LC3-II between bafilomycin A1-treated and untreated NPCs were examined to quantify autophagic flux. Our study indicated that autophagic flux was increased in response to t-BHQ. The NAD^+^-dependent mitochondrial deacetylase sirtuin-3 (SIRT3) is known to regulate cellular metabolism, including adaptation to nutrient stresses, such as fasting and dietary restriction [42]. According to findings of previous studies by Kawamura et al. [43] and Tseng et al. [44], we found that oxidative stress upregulates levels of SIRT3. These results are consistent with our findings. However, the mechanism of change in Sirt3 expression is unknown. Satterstrom et al. [45] conducted a bioinformatics study to identify transcription factor(s) involved in SIRT3 induction. Their analysis identified enrichment of binding sites for Nrf2, which play a role in the expression of mitochondrial function-related genes, including Sirt3 and other genes. t-BHQ treatment also alleviates contrast-induced nephropathy by activating the Nrf2/Sirt3 signaling pathway [34]. Therefore, it is reasonable to assume that t-BHQ resists oxidative stress by inducing a series of genes expressing Nrf2 and increasing Sirt3 to maintain mitochondrial homeostasis and promote mitochondrial autophagy. In the present study, administration of Si-Nrf2 decreased t-BHQ-induced SIRT3 upregulation at both the protein and mRNA levels. Application of Si-Sitr3 also inhibited t-BHQ-induced antiapoptosis and motivated mitophagy, verifying the dominant role of the Nrf2/Sirt3 pathway in t-BHQ-induced treatment in NPCs.

There are some limitations in our study. First, we used the needle puncture model for induction of IVDD, which may not provoke the identical clinical pathology observed in humans. Although this is the most commonly used animal model [46–48], a more appropriate animal model of IVDD needs to be developed for future studies. Second, our study is aimed at exploring the role of t-BHQ in disc degeneration, so the potential effects of t-BHQ on other organs were not shown in our research. However, from previous laboratory studies, we know that t-BHQ protects the liver against ischemia/reperfusion injury and shows no adverse effects in the kidney or other organs in rats [34, 49]. In addition to important organs such as the liver and kidney, intraperitoneal injection of drugs may also have an impact on other organs, and future research on possible side effects of t-HBQ administered systemically remains to be developed.

In conclusion, we demonstrated that apoptosis and mitochondrial dysfunction are activated in TBHP-treated rat NPCs. t-BHQ administration activated mitophagy and restored autophagic flux disruption induced by oxidative stress by triggering the Nrf2/Sirt3 signaling pathway. t-BHQ protected NPCs against apoptosis and the ECM from degradation stimulated by TBHP. Furthermore, we confirmed the therapeutic effect of t-BHQ on rats in the tail disc puncture injury model.

**Figure 8:**
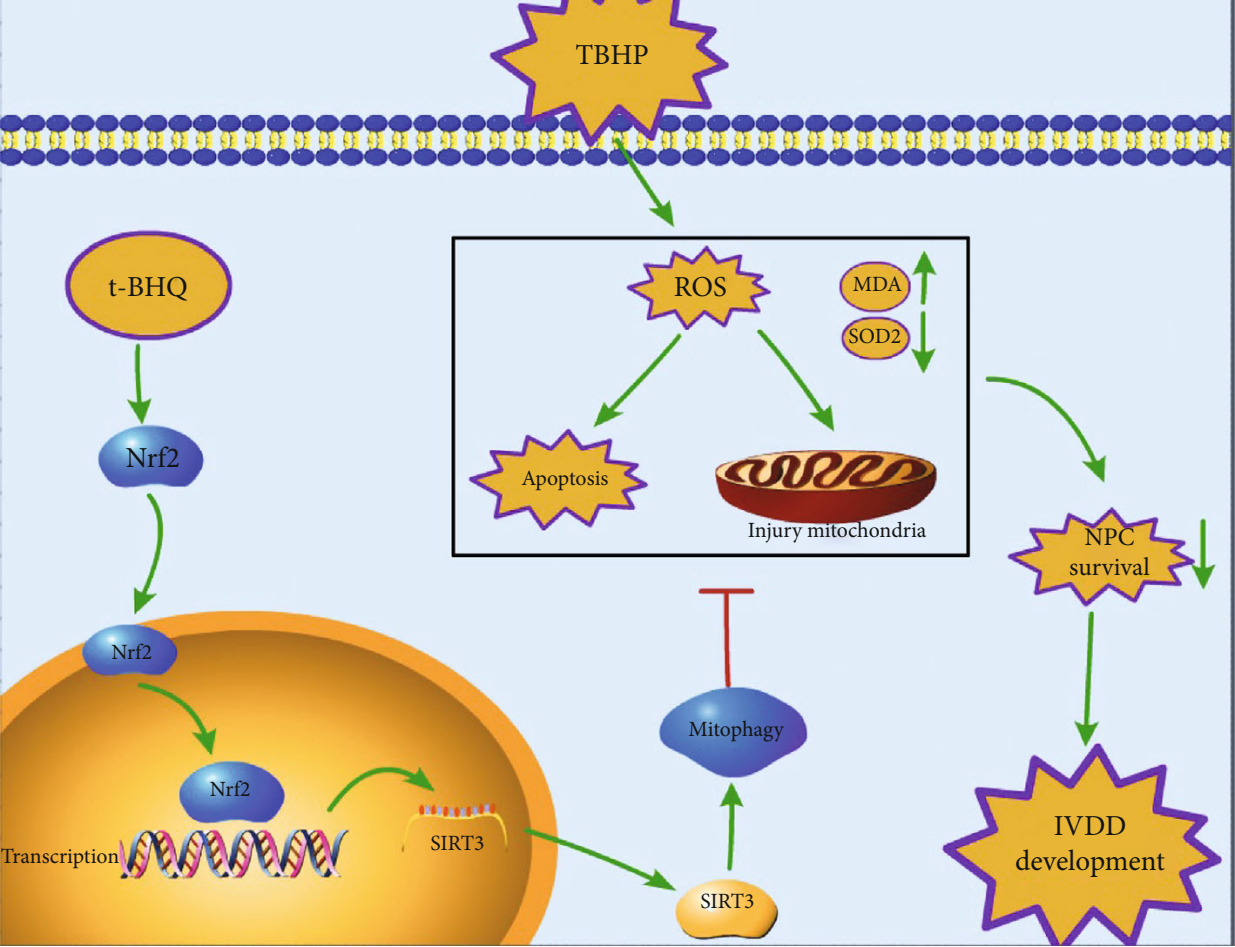
Schematic illustration of the potential protective effects of the t-BHQ and Nrf2/Sirt3 pathway on IVDD.

In conclusion, our study demonstrates the positive role of t-BHQ in IVDD treatment. t-BHQ directly binds to Nrf2, which promotes NRF2 expression and translocation to the nucleus. In addition to the antioxidant and antiapoptotic effects of Nrf2, among many transcriptional downstream genes, higher expression of SIRT3 promotes mitochondrial autophagy to maintain mitochondrial homeostasis and restore blocked autophagic flux, which also alleviates peroxidation stress and apoptosis induced by TBHP in NPCs (Figures 8). Furthermore, t-BHQ and its working mechanism via the Nrf2/Sirt3 pathway contribute to alleviating senescence and uncontrolled extracellular matrix degradation. The schematic illustration shows the protective effects of t-BHQ on NPCs succinctly. Taken together, our findings provide insight into the potential of the Nrf2/Sirt3 pathway and t-BHQ and enrich potential therapeutic strategies against IVDD.

## Data Availability

The data used to support the findings of this study are available from the corresponding author upon request.

## Supplementary Materials

Supplementary Materials Figure S1: effect of t-BHQ on the apoptosis of the TBHP-induced NPCs. Apoptosis was measured using flow cytometry staining with annexin V-FITC/PI.
