# Supplementary material for: Promoting Nrf2/Sirt3-Dependent Mitophagy Suppresses Apoptosis in Nucleus Pulposus Cells and Protects against Intervertebral Disc Degeneration
Source: Oxid Med Cell Longev. 2021 Jun 9;2021:6694964. doi: 10.1155/2021/6694964 (PMC8211502; doi:10.1155/2021/6694964)
Supplement: Supplementary Materials — Figure S1: effect of t-BHQ on the apoptosis of the TBHP-induced NPCs. Apoptosis was measured using flow cytometry staining with annexin V-FITC/PI. [file 6694964.f1.docx]

**Supplementary materials:**

**Figure S1 Effect t-BHQ on the apoptosis of the TBHP induced NPCs.** Apoptosis was measured using flow cytometry staining with annexin V-FITC/PI.
